# Plasticity and Deformation Mechanisms of Ultrafine-Grained Ti in Necking Region Revealed by Digital Image Correlation Technique

**DOI:** 10.3390/nano11030574

**Published:** 2021-02-25

**Authors:** Yonghao Zhao, Yanglin Gu, Yazhou Guo

**Affiliations:** 1 Nano and Heterogeneous Materials Center, School of Materials Science and Engineering, Nanjing University of Science and Technology, Nanjing 210094, China; 15905156972@163.com; 2 School of Aeronautics, Northwestern Polytechnical University, Xi’an 710072, China

**Keywords:** nanostructured metals, plasticity, plastic deformation mechanisms, localized necking, complex stress state

## Abstract

The conventional engineering stress-strain curve could not accurately describe the true stress-strain and local deformability of the necking part of tensile specimens, as it calculates the strain by using the whole gauge length, assuming the tensile specimen was deformed uniformly. In this study, we employed 3D optical measuring digital image correlation (DIC) to systematically measure the full strain field and local strain during the whole tensile process, and calculate the real-time strain and actual flow stress in the necking region of ultrafine-grained (UFG) Ti. The post-necking elongation and strain hardening exponent of the UFG Ti necking part were then measured as 36% and 0.101, slightly smaller than those of the coarse grained Ti (52% and 0.167), suggesting the high plastic deformability in the necking part of the UFG Ti. Finite elemental modeling (FEM) indicates that when necking occurs, strain is concentrated in the necking region. The stress state of the necking part was transformed from uniaxial in the uniform elongation stage to a triaxial stress state. A scanning electron microscopic (SEM) study revealed the shear and ductile fracture, as well as numerous micro shear bands in the UFG Ti, which are controlled by cooperative grain boundary sliding. Our work revealed the large plastic deformability of UFG metals in the necking region under a complex stress state.

## 1. Introduction

Bulk ultrafine-grained (UFG) metals with grain sizes smaller than 1 μm made via severe plastic deformation (SPD) typically have high strength but very low tensile ductility at ambient temperatures [1,2,3]. This is not surprising, as they are derived from excessively cold worked metals. Therefore, during the tensile test, UFG metals do not have the strain hardening stages, and are susceptible to plastic instabilities such as necking due to their low dislocation accumulation capability. In tensile tests, the onset of localized deformation, i.e., necking instability, is predicted by the Considère criterion

(1) $${\left(\frac{\partial \sigma }{\partial \varepsilon }\right)}_{\dot{\varepsilon }}\leq \sigma$$

where *σ* and *ε* are true stress and true strain, respectively, and $\overset{\cdot }{\varepsilon }$ is the strain rate. For the UFG materials, their strength at the right-hand side of Equation (1) is high and the strain hardening rate at the left-hand side of Equation (1) is low, making it easy for premature necking even at a small tensile strain. As a result, their tensile stress-strain curves peak quickly after yielding, and then drop until fracture due to strain localization.

Because the UFG materials have a propensity for strain localization, the low ductility of the UFG materials may limit their applications. Many investigators tried to enhance the ductility of the UFG materials by increasing the storage capacity of dislocation. Those strategies are mostly based on either changing the testing conditions (such as strain rate and/or temperature [4,5]) or designing microstructures (such as formation of bimodal microstructure [6,7], introduction of second phase precipitates [8], etc.). However, not much attention has been paid on the effect of the stress state. Some results have showed that the stress state may also affect the strain hardening behavior [9,10]. Up to now, only a few investigations have focused on the effect of the UFG microstructure on sheet formability, in which the UFG material is deformed in the multiaxial mode along a complex strain path. These studies suggest that even though the UFG sheet materials show very limited deformability in the tensile test, their formability in stretch forming were relatively good [11,12]. However, there is no systematic study on the plasticity and deformation mechanisms of the UFG materials under the complex stress state. Here we will explore the influence of the complex stress state on the mechanical behavior of the UFG materials by focusing on the post-necking deformation during tensile test, which deformed under the complex stress state.

When necking instability occurs, deformation is concentrated in the local part, and the stress state of the necking part was transformed from a uniaxial to multiple axial stress state. Therefore, the necking deformation in tensile testing provides an excellent opportunity for us to reveal the intrinsic deformation behavior of UFG metals under a complex stress state. Due to rapid strain localization and the complex geometry of the neck, it is challenging to determine the real-time actual flow stress and local strain during the whole necking process in tensile tests, which hinder the measurements of real strain hardening ability and plasticity of the UFG materials under a complex stress state [13]. Here, we demonstrated the direct visualization of the necking process by 3D optical measuring digital image correlation (DIC) techniques. The ARAMIS software invented by GOM provides real-time results for multiple measurement positions on the tensile specimen surface. This technology is useful to measure the full strain field and local strain during the whole tensile process, so it was used to calculate the real-time strain and actual flow stress in the necking region.

The objectives of this study are two aspects: first, to explore a new approach that can be used to describe the actual flow stress and strain hardening behavior of the necking region of the UFG materials; and second, to study the mechanical properties and deformation mechanisms of the UFG materials under a complex stress state.

## 2. Materials and Methods

### 2.1. Sample Preparation

Commercially pure Ti (grade 4, 99.405 wt.%) was chosen as a modal material in this investigation, of which chemical compositions are listed in Table 1. The coarse-grained (CG) Ti were obtained by annealing at 680 °C for 1 h in the atmosphere of nitrogen. The Ti rods with a diameter of 10 mm were then processed by the equal-channel angular pressing (ECAP) technique at an ambient temperature using the Bc route for 4 passes to achieve their UFG structure. Route Bc is defined as 90° rotations of the bar in the same direction between each pass. The ECAP die has the internal channel angle φ = 90° and outer angle ψ = 0°. An effective strain of approximately one was imposed per ECAP pass, respectively.

### 2.2. Tensile Test and DIC Technique

**Figure 1:**
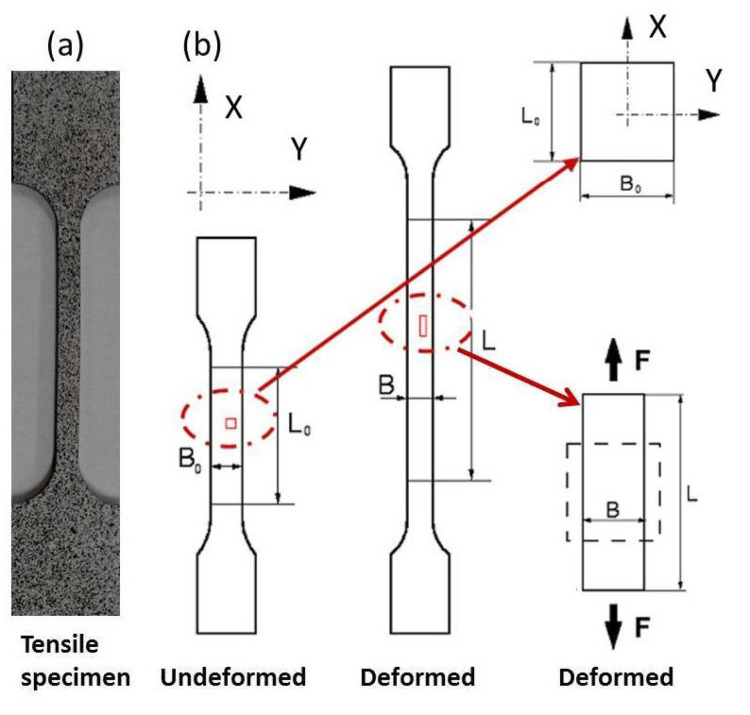
(**a**) Tensile specimen image of which the surface is painted in a random black and white pattern in order to evaluate the strain. (**b**) Schematic representation of 3D optical measuring digital image correlation (DIC) techniques to measure the full strain field and local longitudinal and transversal strains *ε*_X_ = (L − L_0_)/L_0_ and *ε*_Y_ = (B − B_0_)/B_0_, respectively.

For the tensile test, the UFG and CG Ti samples were cut into dog-bone plate shape by the electric discharge machining (EDM) with a gauge dimension of 3 × 1 × 20 mm. Tensile direction was parallel to the ECAP extrusion direction. Tensile tests were run on a ***w + b*** testing machine in quasi-static loading at a strain rate of 10^−4^ s^−1^. In each tensile test, the strain was measured by 3D optical measuring techniques. To evaluate the strain, the surface of the specimen was covered by black and white paints and formed random patterns, as shown in Figure 1a. These patterns were created by spraying a background of flexible, adhesive, matte white paint on a previously degreased specimen. A thin layer of spots of black paint was then sprayed onto the white background [14,15]. Figure 1b schematically represents the measurement theory of local longitudinal and transversal strains (*ε*_X_ = (L − L_0_)/L_0_ and *ε*_Y_ = (B − B_0_)/B_0_), respectively. Two charge coupled device (CCD) cameras with a certain angle were used to take real-time digital images in the gauge length during the entire tensile testing. The recorded images were then analyzed by ARAMIS software to obtain the displacements of the corresponding point by correlating the grey level of both the reference and comparative images. In order to get the strain distribution in the gauge length during the tensile process, the definitions of a facet size, which depends on the pattern applied to the specimen surface, and a facet step, which defines the distance between two facet centre points, are required. Since the gray value of each facet is different, the whole field and local strains can be obtained by tracking the area with the same gray value in the stretching process. Load/stress data were acquired as a function of time from the tensile testing machine, and then imported to the ARAMIS software. The two measurements were correlated to obtain the stress-strain plot of the specimen.

### 2.3. Finite Element Modeling

Finite element modeling (FEM) was used to simulate the triaxial stress state and stress triaxiality *η* in the necking region. The commercial finite element software ABAQUS was used to calculate this, and the analysis method was standard/static. No disturbance was added in the calculation process, and the necking was caused by the geometric size of the sample and the cumulative error of calculation. The geometric dimensions, constraints and loading methods of the tensile calculation models for the UFG and CG Ti were identical, and the same as the experiments. Linear reduced integral unit (C3D8R) was adopted as the element type. The loading mode was displacement loading where one end of the specimen was fixed, while another end was subjected to a velocity load, and the loading direction was consistent with the experimental tensile direction. The total displacements of UFG and CG Ti were 2 mm and 4 mm, corresponding to engineering strains of 10% and 20%, respectively. A trilinear constitutive model was adopted to simulate the tensile curve by inputting the experimental mechanical parameters of the UFG and CG Ti (Table 2 below). The modulus is 106 GPa for Ti.

### 2.4. Microstructure Characterization

Microstructures of the UFG Ti samples were characterized using a transmission electron microscope (TEM). The TEM observations of Ti were carried out on a Philips CM12 microscope operated at 100 kV. To prepare TEM specimens, the UFG C Ti samples were prepared by first mechanically grinding the samples to a thickness of about 50–70 μm, then dimpling to a thickness of about 20 μm, and finally ion-milling to a thickness of electron transparency using a Gatan Precision Ion Milling System with an Ar+ accelerating voltage of 4 kV and a temperature below 35 °C. Important information about ductile damage evolution and fracture mechanisms can be gathered by scanning electron microscopy (SEM).

## 3. Results

### 3.1. Microstructures of the UFG Ti

**Figure 2:**
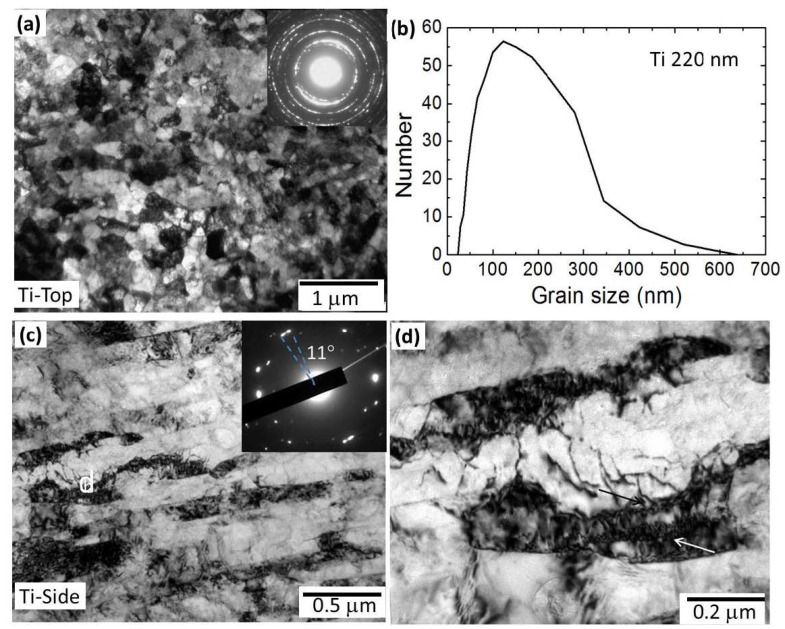
Transmission electron microscope (TEM) images of the UFG Ti from both top (**a**) and side views (**c**,**d**). The insets in (**a**,**c**) have a selected area electron diffraction (SAED) pattern. (**b**) The top-view grain size distribution histogram of the ultrafine-grained (UFG) Ti. (**d**) is the magnified image of the area marked “d” in (**c**).

Figure 2 presents the typical bright-field TEM images for the UFG Ti from both top (radial cross section of the ECAPed rod) and side views (longitudinal section of the ECAPed rod). It is apparent that the microstructures of the UFG Ti are anisotropic with uniform equiaxed grains from top view and elongated grains from side view. The ring-like selected area electron diffraction (SAED) pattern taken from an area with a diameter of 5.4 μm in Figure 2a indicated that grains from top view are randomly orientated, with high-angle grain boundaries. The grains size ranges from about 50 nm to 600 nm, with an average size of about 220 nm (Figure 2c). Lamellar grains with small-angle grain boundaries (approxomately 11° from the SAED pattern) were found in side view, along approximately 30° with ECAP extrusion direction. Moreover, nearly all grains are decorated with dislocations inside. Part of the grain boundaries might be non-equilibrium boundaries [16], as pointed by arrows in Figure 2d. From literature, the non-equilibrium boundaries contain a high density of extrinsic dislocations, which are not needed to accommodate the misorientation across the grain boundary [2]. The above microstructural characteristics of the ECAPed UFG Ti are consistent with the results reported in the literature [17].

### 3.2. Tensile Behavior of the UFG Ti

**Figure 3:**
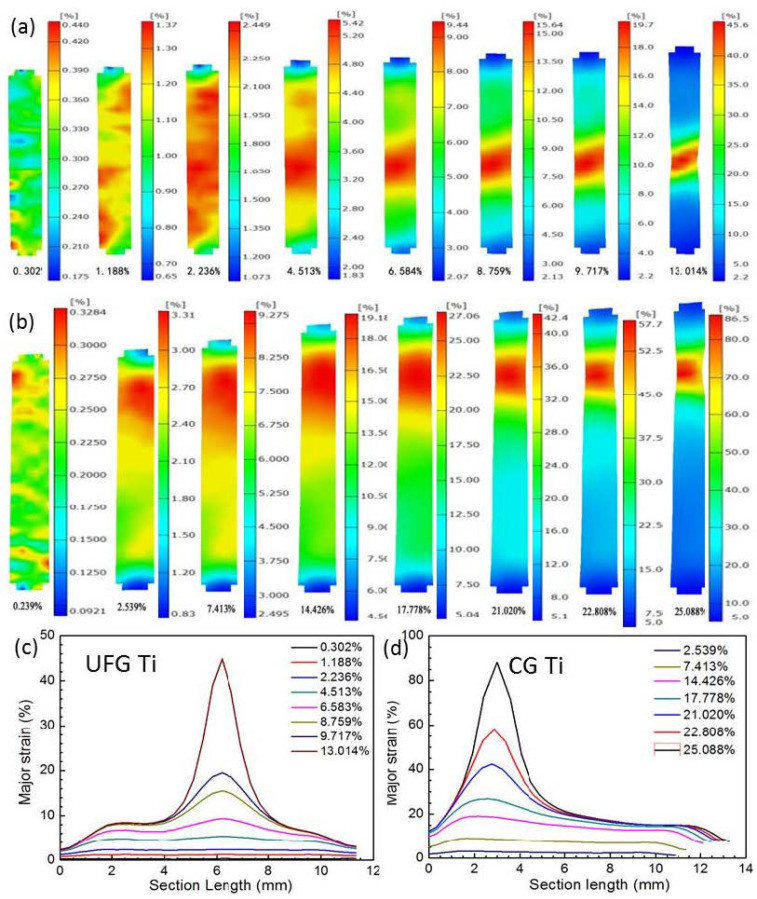
Strain field contour maps (**a**,**b**) and true local major/longitudinal strain *ε*_X_ (**c**,**d**) at eight levels of global engineering strain in the whole gauge part of the UFG (**a**,**c**) and coarse-grained (CG) (**b**,**d**) Ti, respectively.

Figure 3 shows the measured strain field contour maps and true local major strains along tensile axis at eight levels of global major engineering strain *ε* in the whole gauge length of the UFG and CG Ti, respectively. For the CG Ti, at the beginning of loading when *ε* = 2.539%, the specimen was deformed homogeneously throughout the entire gauge length. With increasing *ε* up to 7.413%, the deformation is gradually extended to the whole tensile sample due to strain hardening effect of the CG Ti. When *ε* = 14.426%, the load reaches its maximum, stress concentration was triggered, and deformation became localized. From Figure 2d, one can see the distribution of the true major strain is not uniform in the necking part, but exhibits a peak with maximum value in the necking center. When *ε* = 25.086%, i.e., a major strain near fracture, the maximum true major strain in the necking part can be as high as 90%. Different from the CG Ti, the deformation of the UFG Ti is concentrated in the middle of the tensile specimen from 4.512% global strain until fracture, as shown in Figure 3a. The true major strain evolution of the UFG Ti in Figure 3c further verified the above deformation feature. Moreover, the true major strain in the necking part also exhibits a peak distribution with a maximum value of 45% when *ε* = 13.014% (near the sample broken point). Since the strain continues to evolve in the localized region, ARAMIS software cannot resolve the extremely high strain due to the destruction of the speckles. Therefore, the local strains at the failure initiation site are somewhat higher than the strain obtained by the software at the last frame [18].

**Figure 4:**
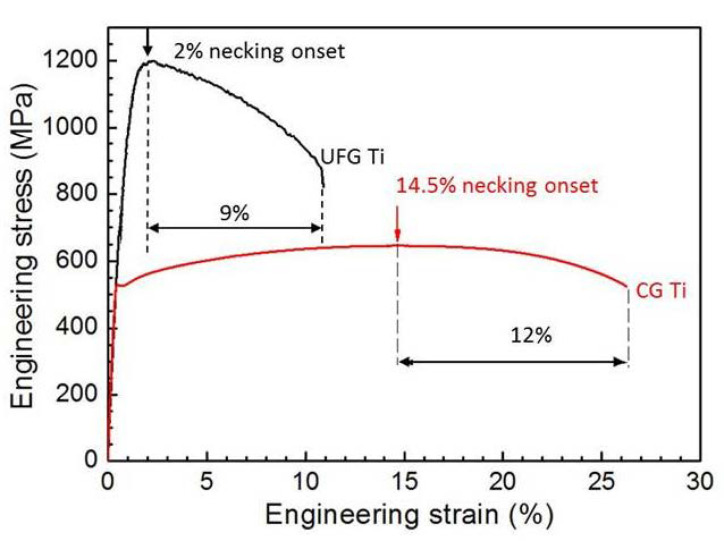
Global engineering stress-strain curves of the UFG and CG Ti.

Combining the global major engineering strain *ε* with the measured load value together, we can easily obtain the traditional engineering stress-strain curves of the CG and UFG Ti, as shown in Figure 4. The CG Ti has a yield strength of 527 MPa, ultimate tensile strength of 640 MPa, uniform elongation of 14.5% and an elongation to failure of 26.5%. The UFG Ti has a high yield strength of 1050 MPa and the ultimate tensile strength of 1200 MPa, but very low uniform elongation of 2% and ductility of 11%. For the UFG Ti, necking occurred quitely after yielding, agreeing with the above measured strain field contour maps in Figure 3. Therefore, the low overall tensile ductility of the UFG Ti was mainly caused by its almost missing uniform elongation. The premature necking instability of the UFG Ti was explained by its nearly null strain hardening capability [19]. The ECAP processing consumed the strain hardening capability by saturating dislocation accumulation, therefore further dislocation accumulation and strain hardening become difficult during subsequent tension. However, different from the huge differences in uniform elongation between the UFG and CG Ti, the post-necking elongation of the UFG Ti (9%) is slightly smaller than that of the CG Ti (12%), hinting a strong deformability of the UFG Ti in the necking part. In addition, after necking onset, the uniform deformation of the whole gauge part of the material changes to the local deformation of the necking region, and the other parts outside the necking region are no longer deformed and enter the “frozen” state, so it is unreasonable to calculate the strain with the whole gauge part as it cannot accurately reflect the local deformation capacity of the material after necking. Or, to put it another way, the measured engineering stress-strain curves could not describe the true stress-strain of the necking part accurately, as it assumes the tensile specimen was deformed uniformly. This causes us to have to re-examine the stress-strain curve of the necking part by 3D optical measuring techniques.

**Figure 5:**
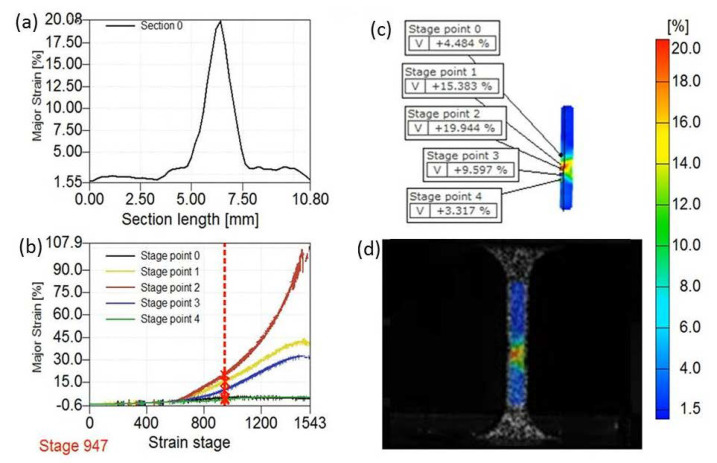
A snapshot from the measured video of the UFG Ti at stage 947. (**a**) True local major/longitudinal strain *ε*_X_ in the whole gauge part. (**b**) True major/longitudinal strain evolutions of five points near necking zone in the UFG Ti during tensile test. (**c**) Positions of the five points near necking zone in the UFG Ti during tensile test in (**b**). (**d**) Image of whole gauge part of the UFG Ti.

To compare the plastic capability of the UFG and CG Ti after uniform deformation, only the necking stage needs to be taken into consideration. ARAMIS software can give the local strain evolution of each point on the tested sample, which is effective to define the necking region. Figure 5 is a snapshot from the measured video of the UFG Ti at stage 947. The DIC measurement videos for the UFG Ti and CG Ti (Videos S1 and S2) were provided in the Supplementary Materials. Figure 5b,c show the real-time true major strain variations of five points with numbers of 0 to 4 on the UFG Ti tensile specimen during the tension. At the beginning, the major strain uniformly increases with the time in the whole gauge length. After a certain instant, however, the strain begins to concentrate within a local part, and the major strain in the local part rises exponentially. The strain of point 2 increases monotonically until fracture, while the strains of points 1 and 3 cease to increase, and even undergo elastic unloading immediately before the final fracture, showing a downward trend. It can be inferred that point 2 is located in the necking instability region, while points 1 and 3 are located in regions outside but close to the necking zone [15]. The actual gauge length for the necking part can be determined by selecting the two points most adjacent to the strain localization zone and defining the region between the two points as the necking region. The neck lengths of the UFG and CG Ti specimens are 1.951 mm and 2.081 mm, respectively. The CG Ti shows slightly higher resistance to the necking deformation due to its higher strain hardening ability, making the necking deformation distribute in a larger range than the UFG Ti.

**Figure 6:**
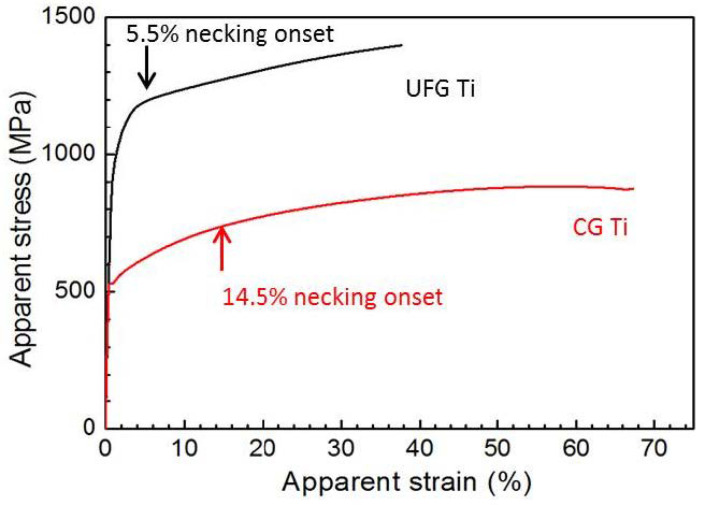
Apparent stress-strain curves of the UFG and CG Ti in the necking region.

The calculated major strain (l − l_0_)/l_0_ based on the actual gauge length of the necking part l_0_ (l is instantaneous length of the necking part) was then called the apparent strain. Obviously, the apparent strain is a global average of the local longitudinal strain in the necking part, and it is more accurate than the nominal engineering strain. In the same way, because the necking region is becoming thinner, the engineering stress, calculated by dividing the load by the initial cross-section area, cannot accurately reflect the real stress change in the necking region [20]. Here, we divided the axial tensile load by the minimum cross-sectional area in the necking region to calculate the apparent stress. Obviously, the apparent stress represents the maximum true stress in the necking region, and is more accurate than the nominal engineering stress. Then we get the apparent stress-strain curves of the UFG and CG Ti in the necking part, as shown in Figure 6. First, for both UFG and CG Ti, the apparent stress increases with increasing apparent strain, suggesting there exists strain hardening during the necking deformation of the UFG Ti. The strain hardening exponent *n* is a parameter used for evaluating strain hardening capability, of which values, simulated by the Hollomon equation [21], are 0.101 and 0.167 for the UFG and CG Ti, respectively. Second, considering the CG Ti has a large uniform tensile strain of ~14.5%, the elongation of the necking region for the CG Ti should be calculated by subtracting the uniform elongation, which is ~52%. The post-necking elongation of the UFG Ti is calculated as 36%, which is greatly larger than traditionally measured value (9%), as listed in Table 2. The post-necking elongation of the UFG Ti is slightly smaller than that of the CG Ti (52%), suggesting a large deformability of the UFG Ti in the necking part. It can be found that despite the great difference between the UFG and CG Ti in their engineering stress-strain curves, there is no big difference between post-necking elongations of the UFG and CG Ti. Considering the deformation under the complex stress state, it can be inferred that the UFG Ti has relatively good plasticity under the multiaxial stress state.

The strain hardening exponent reflects the ability of metal materials to resist uniform plastic deformation. The strain hardening characteristics of the material continue to play a role in the process of resistance to plastic instability. Due to the high strain hardening exponent of the CG Ti, it shows a high resistance to the deformation localization in the necking process, and the necking deformation is distributed in a large range. Therefore, the necking deformation is more diffuse and the neck contour opening is wider. Due to the low strain hardening exponent of UFG Ti, the necking deformation is relatively loose. The development of necking process and the localization resistance of necking deformation are low. This means that the distribution of necking deformation is concentrated, and the opening of neck contour is small. The Poisson’s ratio *ν* of the UFG Ti is slightly smaller than that of the CG Ti, as listed in Table 2, which was caused by the slightly low deformability of the UFG Ti.

### 3.3. Finite Element Modelling (FEM)

Although the DIC technique could measure strain field contour maps in the whole gauge length of the CG and UFG Ti, DIC could not give any information about the stress state and distribution of the specimen in the real experiment, which could only be inferred by observing the stress state in the FEM simulation model before fracture. In the following part, S11, S22 and S33 are referred to as true stress in length, width and thickness directions, respectively. The stress triaxiality *η* is defined as

(2) η=−p/q

(3) $$q=\sqrt{\frac{3}{2}S_{ij}S_{ij}}$$

(4) $$S_{ij}=\sigma_{ij}+p\delta_{ij}$$

(5) $$p=-\frac{1}{3}\sigma_{ii}$$

where *q* is Mises equivalent stress, *p* is hydrostatic pressure and $\delta_{ij}$ is the Kronecker delta. *η* is a physical quantity that characterizes the stress state of the structure, and is usually used to reflect the constraint degree of material deformation. In one-dimensional stress state, *η* = 0.333, and the deviation of *η* from 0.333 indicates that it is a multi-axial stress state.

**Figure 7:**
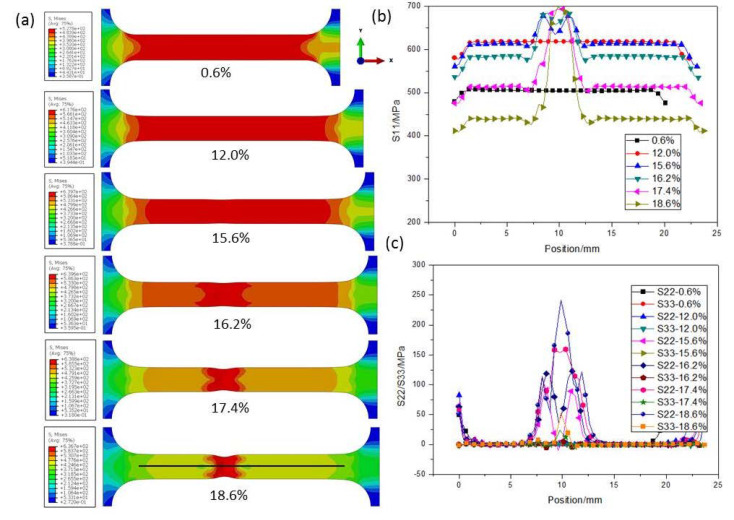
(**a**) The Mises equivalent stress *q* distribution map of CG Ti under different tensile strains. (**b**) Distribution of stress in the tension direction (S11) along the black line in (**a**) of the CG Ti. (**c**) Distributions of stresses in the directions of width (S22) and thickness (S33) along the black line of the CG Ti.

**Figure 8:**
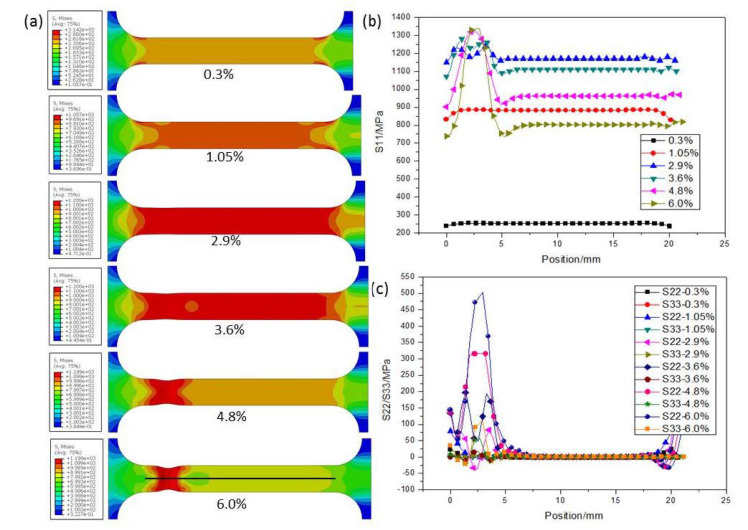
(**a**) The Mises equivalent stress *q* distribution map of UFG Ti under different tensile strains. (**b**) Distribution of stress in the tension direction (S11) along the black line in (**a**) of the UFG Ti. (**c**) Distributions of stresses in the directions of width (S22) and thickness (S33) along the black line of the UFG Ti.

Figure 7a and Figure 8a present the Mises equivalent stress *q* distribution maps of CG and UFG Ti under different tensile strains, respectively. Distributions of stresses in the tension direction (S11) and the directions of width (S22) and thickness (S33) along the black line of the CG and UFG Ti are shown in Figure 7b,c and Figure 8b,c. It can be seen that in the initial stage of drawing, the specimen is in a uniaxial stress state, and there is only stress along the tensile axis, while necking introduces three-dimensional stress state. It should be noted that the axial stress (S11) outside the necking region goes down after stress concentration starts, indicating an unloading effect, which agrees with the test results (Figure 4).

**Figure 9:**
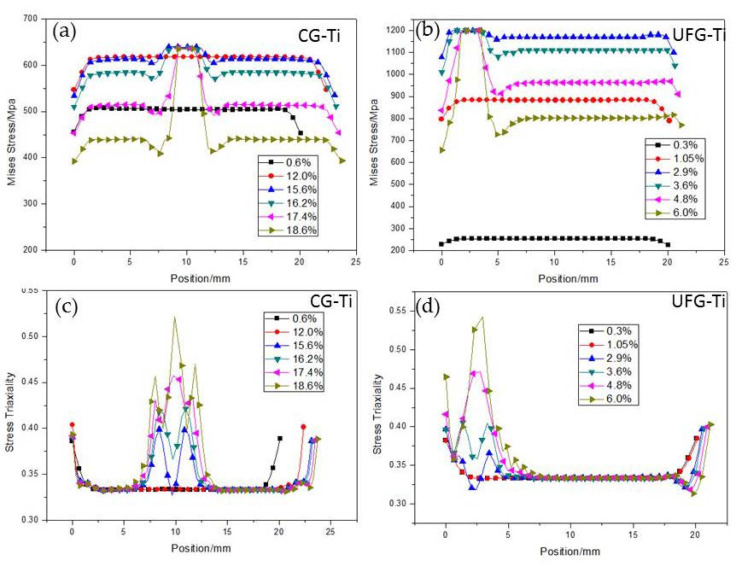
(**a,b**) Mises equivalent stress distributions in the tension direction (S11) along the black line in Figure 7a and Figure 8a of the CG Ti (**a**) and UFG Ti (**b**). (**c,d**) Stress triaxiality distributions along the black lines in Figure 7a and Figure 8a of the CG Ti (**c**) and UFG Ti (**d**).

Mises equivalent stress is usually used to analyze the deformation of materials under a three-dimensional stress state. The *q* boils the complex stress state down to a single scalar number, regardless of the mix of normal and shear stresses. The *q* evolution along the gauge length on the sample surface in the process of tension of both the CG and UFG Ti is given in Figure 9a,b. It can be seen that the *q* of the necking region increases due to the deformation localization, which introduces multi-axial stress state for the local material. This might lead to activation of more slip systems or other deformation mechanisms, which are not active during plastic deformations in the uniaxial stress due to the high critical slip shear stress (CRSS) of UFG Ti. As a result, the inactive slip systems can be activated in a three-dimensional stress state, more grains in the UFG Ti take part in the sliding, which makes the plastic deformation ability of the UFG Ti in the necking region only slightly smaller than that of CG Ti. Figure 9c,d show the stress triaxility *η* distribution along the gauge length on the sample surface of both the CG and UFG Ti. During the tensile process, *η* deviated from 0.33 (uniaxial tension) gradually, and reached the maximum value of 0.525 and 0.55 for CG and UFG Ti, respectively. The stress triaxiality directly affects the fracture strain. Previous studies indicate that smaller stress triaxiality will contribute to the larger fracture elongation [22,23,24] for tensile case. According to the results of FEM, the stress triaxiality of the UFG Ti did not appear to be much different from the CG Ti, which means the difference of the plastic capability under the complex stress state between the UFG Ti and CG Ti is small.

### 3.4. Ductile Fracture Surface

**Figure 10:**
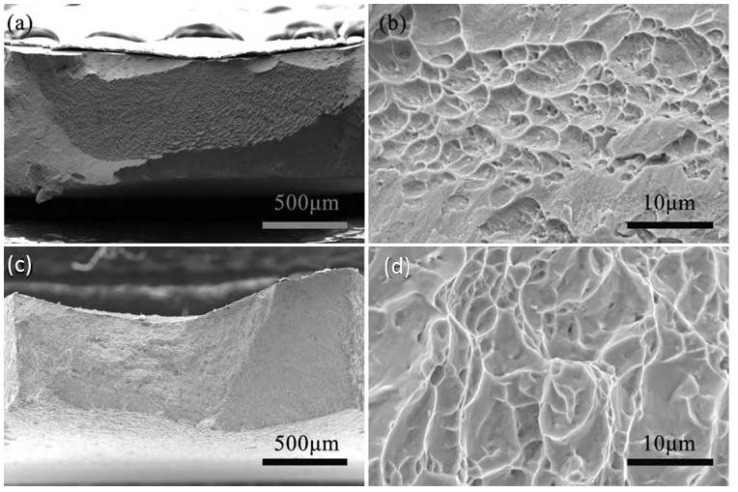
Scanning electron microscopic (SEM) images of the fracture surfaces of the UFG (**a**,**b**) and CG Ti (**c**,**d**). (**a**,**c**) Low magnification. (**b**,**d**) High magnification.

To further understand the tensile properties and build the relationship between microstructures and mechanical behavior, we studied the surface morphology and fracture mode using the SEM. Figure 10 shows SEM images of the macro- and micro-scale fracture surfaces of the UFG and CG Ti samples, respectively. The CG Ti sample fractured via a ductile mechanism with a larger area reduction of fracture surface of 50% and the numerous dimples over the entire fracture surface. From the micro-scale SEM images in Figure 10d, homogeneously distributed honeycomb-like dimples were observed having an average size larger than one micrometer. Moreover, the dimples are elongated due to void nucleation and subsequent coalescence via shear fracture, as revealed below and discussed in Section 4.2.

For UFG Ti, the fracture surface is rough, with a high concentration of uneven concave. A close examination on the concaves in the fracture surface revealed a large number of homogeneously distributed honeycomb-like dimples. Careful inspection showed that the dimples include two types: one has an average size in a range of sub-micrometer, while another has an average size of several micrometers, as shown in micro-scale SEM image in Figure 10b. Although the size of the dimples is smaller compared to CG Ti, they can also provide ability of ductile deformation. In fact, the UFG Ti indeed fractured in a ductile manner, as evidenced by a large area reduction of fracture surface of 41%. The larger fracture area reduction and dimple size of the CG Ti than those of UFG Ti indicates its larger deformability. It is believed that the dimples are initiation sites for fracture. In the literature, Kumar et al. gives three hypothetical mechanisms responsible for void initiation in fully dense UFG materials, and the spacing of these initiation sites determines the dimple size [25].

### 3.5. Shear Fracture and Surface Relief

**Figure 11:**
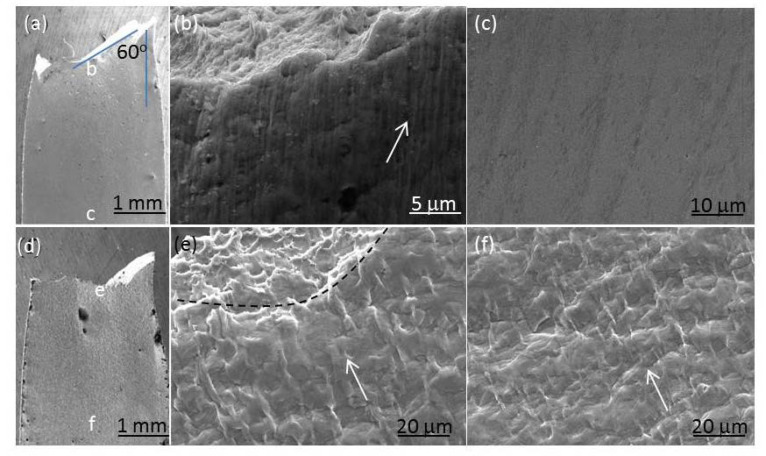
Face-view SEM images of the fractured tensile specimens of the UFG (**a**–**c**) and CG Ti (**d**–**f**). (**b**,**c**) are the magnified images of the necking region near the fracture marked as “a” and the uniform deformed region marked as “c” in (**a**), respectively. (**e**,**f**) are the magnified images of the regions marked as “e” and “f” in (**d**), respectively. The black dashed line in (**e**) shows the boundary between the fracture surface (upper part) and tensile specimen plate surface (lower part).

Figure 11 shows the SEM images from the side-view of the CG and UFG Ti, respectively. The UFG sample failed in a shear fracture mode with a shear fracture angle *θ* (the angle between the fracture surface and tension axis) of about 60°. However, the CG Ti fractured in a normal way, with *θ* = 90°. During the uniform deformation, the strain path is along the direction of the maximum shear stress, i.e., the shear angle is 45°. When necking occurs, the maximum strain path was changed, and the fracture angle will be slightly increased. The deviated shear fracture angle from 45° of the CG and UFG Ti indicated again that necking occurs in both samples, and the CG Ti exhibits a greater degree of deviation due to its higher strain-hardening ability. From fracture mechanics, the larger shear fracture angle than 45° of the CG and UFG Ti indicates that the fracture behavior is controlled by both normal stress and shear stress on the shear fracture surface [20], as calculated and discussed in Section 4.2 in detail.

To study mechanisms operating during plastic deformation under the complex stress state, the surface relief of pre-polished fractured specimens was carefully inspected in SEM. Figure 11b,c,e,f shows the micro-scale surface relief SEM images from face-view of the CG and UFG Ti. For the CG Ti, the surface relief in the homogeneous plastic deformation area is similar to the necking region, revealing that CG Ti was deformed by dislocation slip under both uniaxial and multi-axial stress states (Figure 11e,f). For the CG Ti, at the initial stage of deformation, grains with maximum shear stress on slip systems begin to slide first. The sliding of dislocations is then blocked and accumulated by GBs, forming a ridge at GBs. With the stress increase, multiple dislocation slips are activated and slide further in the deformed grains, causing the increase of stress concentration at the boundary. The deformed CG grains are then elongated with the help of numerous dislocation slip bands. When the stress reaches a certain degree, the dislocations slip systems in neighboring grains are excited. The slip bands in the adjacent grains are blocked at GBs and forming ridges at GBs (pointed by white arrow in Figure 11e,f). Because the critical slip shear stress of CG Ti dislocation is small, the deformation is easy to transfer from one grain to another, so that more and more grains participate in the sliding, resulting in the large plastic deformation of the sample.

For the UFG Ti, the surface relief in the necking area has much higher roughness than the uniform deformed region (Figure 11b,c). Careful observation on the sample surface revealed the numerous rough localized plastic deformation markings or traces parallel with each other in the necking region on the fractured specimen, as pointed by white arrow in Figure 11b. Several published studies reported similar deformation traces in UFG Al [26], 6082 Al alloys [27], Ni and Cu [28], which were described as microscopic or mesoscopic shear bands or shear planes. From Figure 8b, the distances between shear bands are several micrometers, and the lengths of the shear bands extend from several micrometers to several hundred micrometers. The size of the shear bands is much larger than the grain size of the UFG Ti (~220 nm), suggesting cooperative GB sliding may involve in the plastic deformation, which was further revealed below and discussed in Section 4.3.

## 4. Discussion

A large number of previous literature shows that the plastic deformation ability of UFG material is much lower than that of its CG counterparts. However, the conventionally measured engineering stress-strain curves could not describe the true stress-strain of the necking part accurately, as it assumes the tensile specimen was deformed uniformly. In this work we used the DIC technique to accurately measure the full strain field, as well as local strain during the whole tensile process. Our results indicated that the plastic deformability in the necking part of the UFG Ti is only slightly smaller than that of CG counterpart. Postmortem observation revealed the tendencies of shear and ductile fracture, and numerous micro shear bands in the UFG Ti. In the following, we will discuss the abovementioned results one by one from the angle of deformation mechanisms and fracture mechanics.

### 4.1. Necking

#### 4.1.1. Nonlinear Section Shrinkage and Influence Factors

Necking is the phenomenon of specimen cross-sectional reduction during tension and is a joint action of hardening and weakening mechanisms. During uniform deformation before necking, the specimen elongation and section shrinkage are linear, and conform to the condition of volume invariability. After necking, however, the specimen elongation is only borne by the necking region. In order to maintain the continuity of the specimen under the control of the chuck, the necking region must be accelerated to shrink, and the section closer to the center of necking region will shrink faster, which is called nonlinear section shrinkage [20]. As revealed by DIC results in Figure 5 and Figure 6b, the *ε*_X_ in the necking region of the CG Ti increases exponentially after 800 s of loading.

The influence factors of necking include intrinsic characteristics of materials, such as the strain hardening exponent *n* and strain rate sensitivity *m*, and external deformation conditions, such as temperature, strain rate, stress states, etc. After necking, the strain hardening and rate hardening mechanisms still work, and further resist the necking process. The larger *n* is, the greater the deformation resistance is. The strain rate sensitivity *m* indicates that the deformation resistance increases with the increase of strain rate. The higher strain rate leads to the increases of dislocation density and dislocation movement rate, i.e., the increase of work hardening degree. Because both strain and strain rate at necking increase, large *n* and *m* values will result in the further reinforcement of deformation resistance in the necking region, causing the unreinforced part continues to deform. The positive *n* value (0.101 and 0.167) and large *m* (0.02 and 0.04 [17,29]) in the UFG and CG Ti will expand the necking process and delay fracture, i.e., enhance the plastic deformability of the necking region.

#### 4.1.2. Diffuse and Local necking

**Figure 12:**
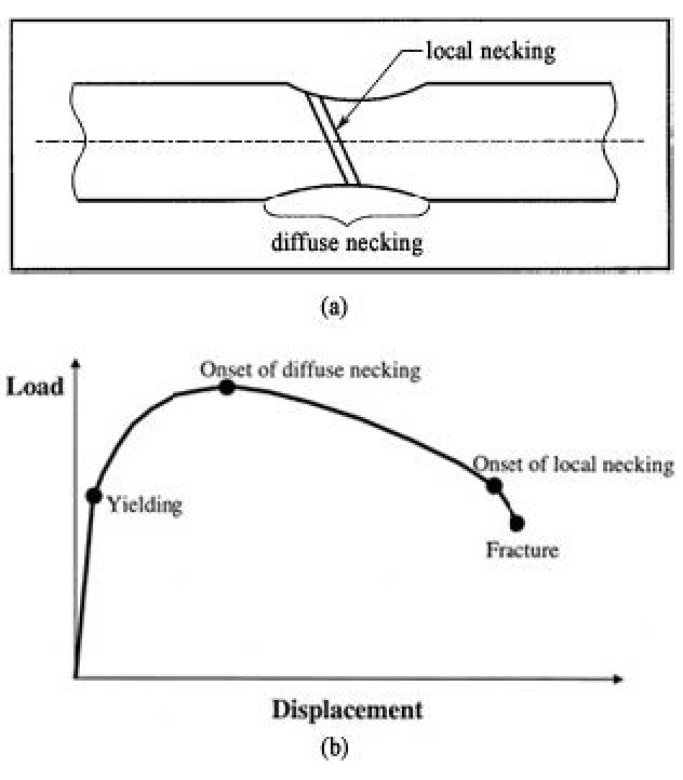
Schematic representation of the necking process of plate tensile specimen. Two stages of diffuse and local necking occurred during fracture. (**a**) Schematic representation of the necking process. (**b**) Tensile curves to show yielding, onset of diffuse and local necking as well as fracture.

For plate tensile specimens, fracture includes two stages: diffuse and local necking [30,31], as shown in Figure 7a. Because of the internal defects in the material, when the load reaches a critical value in the tensile process, the local uneven plastic deformation first appears in the weak area with internal defects, and produces strain strengthening. As a result of the strain strengthening in the local area, the load must be increased to make the other neighboring weak areas deform locally. The necking diffuses on the specimen as the tensile process proceeds. Once the diffuse necking is formed, the material will change from uniform deformation to localized deformation, resulting in strain localization and a complex stress state. After diffuse necking, local necking usually occurs and eventually leads to fracture. The local necking is usually a narrow banded area, at an angle to the tensile axis. Once the local necking is formed, the material will fracture quickly. For the plate tensile specimens with large ratio of width to thickness and low strain hardening exponent, the final fracture direction generally occurs along the width direction, as observed in Figure 12. Conversely, the final fracture direction occurs along the thickness direction.

### 4.2. Shear fracture angle

**Figure 13:**
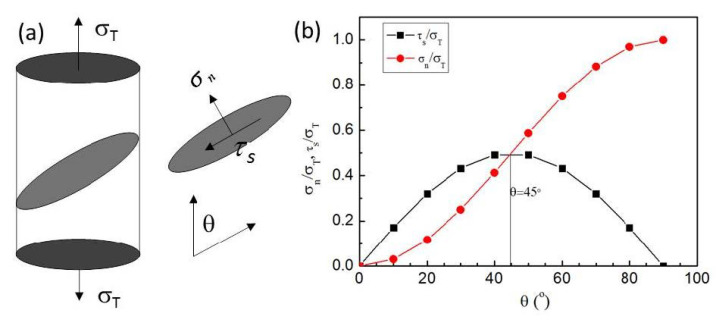
(**a**) Schematic representation of the normal and shear stresses (*σ_n_* and *τ_s_*) on the shear plane with a shear angle *θ* under tensile stress *σ_T_*. (**b**) The variations of the normal and shear stresses against the shear angle *θ*.

For the plate tensile specimens, the final fracture mode is usually along the width or along the thickness direction with a shear angle under the combined action of normal stress and shear stress. At the same time, the shear angle is also related with the intrinsic plastic deformation ability of material. As illustrated by the graphic representation in Figure 13a, if the tensile stress *σ_T_* is imposed on the sample, the normal and shear stresses (*σ_n_* and *τ_s_*) on the shear plane with a shear angle *θ* can be expressed as:

*σ_n_* = *σ_T_* sin^2^*θ* (6)

*τ_s_* = *σ_T_* sin *θ* cos *θ* (7)

It can be seen from Figure 11b that when *θ* is less than 45°, *σ_n_* is less than *τ_s_*. When *θ* = 45°, *σ_n_* = *τ_s_*, and the fracture is generated under the maximum shear stress, which conforms to the Tresca fracture criterion; when *θ* is greater than 45°, *σ_n_* > *τ_s_*, and the fracture is produced under the joint action of shear stress and normal stress. When *θ* = 90°, *σ_n_* reaches its maximum and *τ_s_* = 0, the fracture follows the maximum normal stress criterion.

Because the intrinsic shear strength of materials *τ_0_* is smaller than the cleavage normal breaking strength *σ_0_*, during the uniform deformation, the strain path is along 45°, i.e., the loading direction of the maximum shear stress. If there is no necking in the tensile process, the final *θ* is close to 45°. When necking occurs, the plastic deformation path of the material was changed, and the fracture angle will be increased over 45° [23,24]. The fracture angle and dimple size increase with the necking degree. For plate tensile specimen of ductile materials, shear fracture is the main fracture mode, as the necking cannot reach the ideal degree before fracture. The deviated shear fracture angle from 45° of the CG and UFG Ti indicated that necking occurs in both samples, and the CG Ti exhibits a slightly greater degree of the necking process, due to its higher strain hardening ability.

To better understand the shear angle and the shear fracture mechanism, Zhang et al. [20] proposed a unified tensile fracture criterion, as seen below:

(8) $${\left(\frac{\sigma_{n}}{\sigma_{0}}\right)}^{2}+{\left(\frac{\tau_{n}}{\tau_{0}}\right)}^{2}=1$$

According to the unified tensile fracture criterion, *τ_0_* and *σ_0_* can be calculated by the following equations

(9) $$\sigma_{T}=2\tau_{0}\sqrt{1-\alpha^{2}}$$

(10) $$\theta_{T}=\frac{\pi }{2}-\frac{1}{2}\arctan\left(\frac{\sqrt{1-2\alpha^{2}}}{\alpha^{2}}\right)$$

(11) $$\alpha =\frac{\tau_{0}}{\sigma_{0}}$$

where the ratio *α* is a fracture mode factor controlling the macro-scale fracture modes of a material. It is suggested that when 0 < *α* < 2^0.5^/2, the shear fracture angle *θ* is in the range of 45–90° [20].

According to the unified tensile fracture criterion, the ratios *α* of the CG and UFG Ti were calculated as 0.71 and 0.45, respectively. Their *σ_0_* and *τ_0_* were 673 MPa/478 MPa and 2209 MPa/994 MPa, respectively. As shown in Table 3, *σ_0_* and *τ_0_* of the UFG Ti are larger than those of the CG Ti. The proportion of *σ_0_* to *τ_0_* in the UFG Ti is also larger than that in the CG Ti, resulting in a smaller ratio *α* and a smaller shear fracture angle. It has been demonstrated that the ECAP process can effectively change the fracture mode from normal fracture to shear fracture with different shear fracture angles [32].

### 4.3. Deformation Mechanisms

Microstructure analysis found that stress mode does not affect the plastic deformation mechanisms of CG Ti much, which is mainly dislocation glide (Figure 6e,f), while does affect those of the UFG Ti (Figure 6b,c). The UFG Ti under uniaxial stress did not exhibit much deformation, while under triaxial stress in the necking region showed numerous micro shear bands. The underlying deformation mechanisms include GB sliding. In the last several decades, numerous investigations of experiments, molecular dynamic (MD) simulations and other modeling efforts on deformation mechanisms of the nanocrystalline and UFG materials have revealed GB-mediated deformation such as grain rotation [33], GB sliding [34], GB diffusion [35] and stress-driven GB migration [36], besides the conventional slip of lattice dislocations [37,38,39]. Grain coalescence or growth usually occurred as a result of the above GB-mediated deformation [40]. In the sections that follow, we discuss each one within the context of the results described herein.

#### 4.3.1. Grain Boundary Sliding

At elevated temperatures, GB sliding, i.e., individual grains displaced with respect to each other along their mutual boundaries as a consequence of an external stress, is a well-established deformation process for CG materials [41]. The sliding of individual grains is accommodated either through the intra-granular dislocation slip or through the diffusional flow of vacancies [41].

The GB sliding of the UFG materials has been reported to occur even at room temperatures [34], as of the enhanced diffusion kinetics and/or stress-driven GB migration [36]. For the former reason, as suggested by literatures, even at room temperature, diffusion can play an important role in the plastic deformation of the UFG metals and alloys. The increased volume fraction of GBs promotes the GB diffusion processes due to enhanced diffusivity [42]. Moreover, high fractions of high-angle GBs and non-equilibrium GBs with many extrinsic dislocations lying in narrow regions adjacent to the GBs have been frequently reported in the UFG materials. These boundaries and the associated high dislocation densities are probable to provide easy diffusive paths for the local re-arrangements needed to form the GB sliding. For the latter reason, stress-driven GB migration is also revealed as an athermal activation process by some in situ TEM observations [23]. The GB sliding is even observed to occur at very low temperatures, such as at liquid nitrogen temperature [43].

#### 4.3.2. Cooperative Grain Boundary Sliding and Micro Shear Bands

Hahn et al. [44] proposed a theoretical model for the deformation of the UFG materials, i.e., formation of mesoscopic glide planes based on the GB sliding. For the UFG materials, the high volume fraction of the GBs was argued to provide an opportunity for the formation of long planar inter-faces by stretching over many grains and resulting in a macroscopic sliding over individual grain dimensions. Moreover, a cooperative GB sliding of the UFG materials was observed at ambient temperatures by in situ SEM and TEM observations [33]. In addition, the above mesocopic shear planes and/or cooperative GB sliding are also revealed by MD and modeling computer simulations [34], as well as experiments [26]. A cooperative GB slide of a series of the UFG grains was observed in UFG Fe, Cu, Al, 6061 Al and Ni [26,27,28].

**Figure 14:**
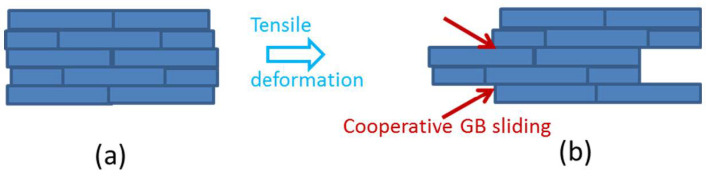
Schematic diagram of the deformation mechanism of the UFG Ti under the complex stress state. (**a**) Initial microstructures. (**b**) Tensile deformed microstructures with cooperative GB sliding.

In CG materials, the initial dimensions of micro shear bands are significantly smaller than the coarse grain size, as they are formed within a coarse grain. However, the micro shear bands of the UFG metals are different from those seen in CG materials. The micro shear bands in CG materials grow by spreading into neighboring grains across GBs, and eventually form macro shear bands. Such a relationship between the grain size and the dimensions of micro shear band does not apply to the UFG metals. Therefore, those shear bands in UFG metals, whose width/grain size ratio falls in the range of 1–10, are defined as micro shear bands. They are distinct from the macro shear bands, which spread across the entire specimen cross-section, form a fracture surface and result in failure. The cooperative GB sliding, contributing the high plastic deformation of the UFG Ti in the necking region under a complex stress state, is schematically shown in Figure 14.

## 5. Conclusions

In summary, the tensile testing with optical measuring DIC techniques is a powerful tool to study the deformation process during the tensile process. It can uncover mesoscopic deformation flow and reveal the details of the strain evolution on surface deformation, which is the footprint of underlying deformation mechanisms. In addition, SEM and TEM techniques were employed to systematically investigate intrinsic plasticity and deformation mechanism of UFG Ti at localized necking parts with a complex stress state. The detailed results are the following:

1. The UFG Ti with an average grain size of 220 nm was achieved by ECAP.
2. DIC measurements indicated that for both UFG and CG Ti, the apparent stress increases with increasing apparent strain in the necking region. Strain hardening exponents *n* are 0.101 and 0.163 for the UFG and CG Ti, respectively. The elongation of the UFG Ti in the necking region is 36%, slightly smaller than that of the CG Ti (52%), suggesting a large deformability of the UFG Ti in the necking part.
3. FEM indicates that when necking occurs, strain is concentrated in the necking region. The stress state of the necking part was transformed from uniaxial in uniform elongation stage to triaxial stress state.
4. SEM study of fracture surface morphology of the UFG Ti revealed the tendencies of shear and ductile fracture with a shear angle of 60°, and numerous homogeneous micro shear bands under triaxial stress state near fracture zone which were controled by cooperative GB sliding.

## Figures and Tables

**Table 1:** Measured chemical composition (wt. %) of the as-received commercially pure Ti (grade 4) by Inductive Coupled Plasma Emission Spectrometer and ONH Analyzers.

**Elements wt.%**	**Fe**	**C**	**N**	**O**	**Ti**
	0.2	0.05	0.005	0.34	99.405

**Table 2:** Lists of yield strength *σ_0.2_*, ultimate tensile strength *σ_UTS_*, uniform elongation *ε_ue_*, post-necking elongation *ε_pe_*, elongation to failure *ε_ef_*, post-necking elongation *ε_pe_*, strain hardening exponent *n*, apparent elongation at necking *ε_ae_* and gauge length of necking region *l_0_* as well as Poisson’s ratio *ν* of the UFG and CG Ti.

	*σ_0.2_*, MPa	*σ_UTS_*, MPa	*ε_ue_*, %	*ε_ef_*, %	*ε_pe_*, %	*n*	*ε_ae_*, %	*l_0_*, mm	*ν*
CG	527	640	14.5	26.5	12	0.167	52	2.081	0.33
UFG	1050	1200	2	11	9	0.101	36	1.951	0.31

**Table 3:** Lists of fracture strength *σ_F_*, shear fracture angle *θ*, ratio *α*, average critical normal fracture stress *σ_0_* and average critical shear fracture stresses *τ_0_* of the UFG and CG Ti.

Samples	*σ_F_* (MPa)	*θ* (°)	*α*	*σ_0_*, MPa	*τ_0_*, MPa
CG Ti	850	90	0.71	673	478
UFG Ti	1400	60	0.45	2209	994

## Data Availability

Data are available from the corresponding author (yhzhao@njust.edu.cn). Supplementary Video is available online for this paper.

## Supplementary Materials

The following are available online at https://www.mdpi.com/2079-4991/11/3/574/s1, Video S1: The DIC measurement video for the UFG Ti; Video S2: The DIC measurement video for the CG Ti.
